# Independent and combined associations between fast-food outlet exposure and genetic risk for obesity: a population-based, cross-sectional study in the UK

**DOI:** 10.1186/s12916-021-01902-z

**Published:** 2021-02-15

**Authors:** Thomas Burgoine, Pablo Monsivais, Stephen J. Sharp, Nita G. Forouhi, Nicholas J. Wareham

**Affiliations:** 1 UKCRC Centre for Diet and Activity Research (CEDAR), MRC Epidemiology Unit, University of Cambridge School of Clinical Medicine, Box 285 Institute of Metabolic Science, Cambridge Biomedical Campus, Cambridge, CB2 0QQ UK; 2 Present address: Department of Nutrition and Exercise Physiology, Elson S. Floyd College of Medicine, Washington State University, Spokane, Washington, USA

**Keywords:** Fast-food outlet exposure, Genetic predisposition, Interaction, Body weight, Obesity

## Abstract

**Background:**

Characteristics of the built environment, such as neighbourhood fast-food outlet exposure, are increasingly recognised as risk factors for unhealthy diet and obesity. Obesity also has a genetic component, with common genetic variants explaining a substantial proportion of population-level obesity susceptibility. However, it is not known whether and to what extent associations between fast-food outlet exposure and body weight are modified by genetic predisposition to obesity.

**Methods:**

We used data from the Fenland Study, a population-based sample of 12,435 UK adults (mean age 48.6 years). We derived a genetic risk score associated with BMI (BMI-GRS) from 96 BMI-associated single nucleotide polymorphisms. Neighbourhood fast-food exposure was defined as quartiles of counts of outlets around the home address. We used multivariable regression models to estimate the associations of each exposure, independently and in combination, with measured BMI, overweight and obesity, and investigated interactions.

**Results:**

We found independent associations between BMI-GRS and risk of overweight (RR = 1.34, 95% CI 1.23–1.47) and obesity (RR = 1.73, 95% CI 1.55–1.93), and between fast-food outlet exposure and risk of obesity (highest vs lowest quartile RR = 1.58, 95% CI 1.21–2.05). There was no evidence of an interaction of fast-food outlet exposure and genetic risk on BMI (*P* = 0.09), risk of overweight (*P* = 0.51), or risk of obesity (*P* = 0.27). The combination of higher BMI-GRS and highest fast-food outlet exposure was associated with 2.70 (95% CI 1.99–3.66) times greater risk of obesity.

**Conclusions:**

Our study demonstrated independent associations of both genetic obesity risk and neighbourhood fast-food outlet exposure with adiposity. These important drivers of the obesity epidemic have to date been studied in isolation. Neighbourhood fast-food outlet exposure remains a potential target of policy intervention to prevent obesity and promote the public’s health.

**Supplementary Information:**

The online version contains supplementary material available at 10.1186/s12916-021-01902-z.

## Background

As a modifiable factor associated with elevated risk of type 2 diabetes [1], cardiovascular disease [2], some cancers [3], and all-cause mortality [4], obesity is a global public health priority. In England, high body weight is one of the leading drivers of mortality and morbidity [5], and obesity costs the National Health Service £6.1bn per year [6]. One of the greatest challenges to addressing this public health problem at a population level is that obesity is a complex, multifactorial condition, including biological and social determinants.

Among biological determinants, genetic epidemiological studies have firmly established the role of genetics in the development of obesity. Genome-wide association studies (GWAS) have been able to identify individual genetic loci associated with obesity and common metabolic diseases [7]. For example, single-nucleotide polymorphisms (SNPs) in the *FTO* gene are associated with higher risk of obesity and type 2 diabetes [8], while SNPs in the *MC4R* gene are associated with increased fat mass and obesity risk [9]. The most recent GWAS have observed SNPs at 97 loci that can collectively indicate an individual’s genetic predisposition to obesity [10], although the level of variance explained is typically less than 2% [11].

The rise of obesity in the last 40 years has also been attributed to changing lifestyles [12], for example, declining physical activity levels and diets increasingly composed of processed, energy-dense foods, which can promote weight gain [13]. Food outlets selling these types of food, typically fast-food outlets, have become ubiquitous in neighbourhood environments [14, 15], and the potential of such access to influence dietary choices and long-term weight status is increasingly recognised [16]. Although some studies report contradictory or null findings, a growing number of cross-sectional and longitudinal studies in the UK and USA indicate that exposure to higher densities of fast-food outlets is associated with higher intake of energy-dense fast foods, higher body weight, and greater odds of obesity [17–22]. However, none of these studies have been able to account for individual-level genetic predisposition to obesity.

Moreover, while there is strong evidence for both genetic and environmental drivers of obesity, to date there is little understanding of how these two types of determinants may interact. *FTO*, *MC4R*, and other SNPs have been associated with appetitive phenotypes including disinhibited eating and preferences for high-fat or energy-dense foods [23], which may be enabled in neighbourhood environments with higher densities of fast-food outlets. Therefore, we investigated the associations of genetic obesity predisposition and objectively-measured neighbourhood fast-food outlet exposure, independently and in combination, with body weight, in a population-based sample of UK adults. Motivated to understand a possible interaction, we also explored how associations between fast-food outlet exposure and body weight might be modified by genetic susceptibility to obesity.

## Methods

### Study population

We used data from the Fenland Study, a population-based cohort of 12,435 adults aged 29–64 years in Cambridgeshire, UK. Study recruitment was from general practice lists across Cambridge, Ely, and Wisbech, conducted by the University of Cambridge MRC Epidemiology Unit. Data were collected from 2005 to 2015. Participants completed general questionnaires related to their lifestyle, medical history, and home address. Weight and height were measured to a standardised protocol by trained researchers, and the participants provided blood samples for genotyping. All study procedures were approved by the Health Research Authority National Research Ethics Service Committee East of England-Cambridge Central.

### Assessment of neighbourhood fast-food outlet access

Data on food outlet locations were sourced from local councils throughout the study area in 2011 (approximately half-way through participant data collection). Fast-food outlets were classified as shown in Additional file 1: Table S1. These food outlets were geocoded at the postcode level using a geographic information system (ArcGIS 10, ESRI). Participant home addresses were also geocoded using postcodes. Using established methods [19, 22, 24], home ‘neighbourhoods’ were characterised as 1-mile straight-line radius (circular) buffers. The number of fast-food outlets was summed within neighbourhoods and categorised using quartiles.

### Genotyping

Recent genome-wide association studies have identified 97 SNPs related to BMI [10]. These SNPs represent obesity susceptibility loci, including those near or in the well-characterised BMI-related *FTO* and *MC4R* genes. The genotyping procedure used here and method of imputation for SNPs not directly genotyped have been described in detail elsewhere [25, 26].

### Genetic obesity predisposition score

Our genetic risk score for BMI (BMI-GRS) was derived from 96 of the 97 BMI-associated SNPs [25]. Each individual has 0, 1, or 2 copies of the BMI-increasing allele at each SNP, with BMI-GRS calculated by summing the number of alleles across all 96 variants. Scores were then weighted by the strength of association of each identified SNP with BMI, with higher scores indicating a greater predisposition to obesity. Participants were dichotomised at the median BMI-GRS (2.29) to define low- and high-risk groups [27].

### Assessment of body weight and obesity

We defined two primary outcomes: body mass index (BMI, kg/m^2^), calculated from measured height and weight, and weight status (overweight: 25 ≥ BMI < 30; obese: BMI ≥ 30).

### Statistical analysis

We used multivariable linear, and multinomial logistic regression models to examine associations between each of home neighbourhood fast-food outlet count (quartiles) and genetic risk (low, high), with BMI (kg/m^2^) and risk of being overweight (25 ≥ BMI < 30) and obese (BMI ≥ 30). Adjusted models included the following covariates theoretically determined a priori: age, sex, household income (< £20,000, £20,000–£40,000, > £40,000), highest educational attainment (≤ 11 years of education, 12–13 years, > 13 years), car access, smoking status (never, current, or ex), physical activity energy expenditure (kJ/kg/day, measured using individually calibrated combined acceleration and heart rate sensors (Actiheart, CamNtech) worn for up to 6 days) [28], and number of supermarkets belonging to major UK chains in the home neighbourhood. To establish independent associations, both fast-food outlet exposure and BMI-GRS models were mutually adjusted.

We tested for evidence of interaction (fast-food outlet exposure quartiles x BMI-GRS *z*-scores) using an *F*-test for BMI and likelihood ratio test for weight status. We estimated means and 95% CIs for BMI in high and low genetic risk groups, from stratified adjusted linear regression models.

We also cross-classified each participant into one of eight groups based on the combination of neighbourhood fast-food outlet exposure (four levels) and genetic risk (two levels) and used multivariable logistic regression with a single reference category (least exposed to fast-food outlets, low genetic risk) to estimate the combined associations of neighbourhood fast-food outlet exposure and genetic risk on the likelihood of being overweight and obese (both vs normal weight, i.e. BMI < 25).

This was a complete case analysis, with the sample restricted to those with complete data across all covariates and outcomes of interest (Additional file 1: Fig. S1). The final analytic sample size was 10,798, remaining representative of the wider Fenland Study cohort across key variables (Additional file 1: Table S2). A two-sided α level of 0.05 was used to test for statistical significance throughout. Data were analysed in 2019–2020 using Stata 14.2 (StataCorp LP., Texas).

## Results

### Sample characteristics

Descriptive statistics, overall and stratified by high and low genetic risk, are presented in Table 1. The sample had a mean age of 49 years and was 47% men, with a mean BMI of 26.9 kg/m^2^, and 21.7% of the sample were classed as obese. Participants were exposed to an average of 9 fast-food outlets in their home neighbourhood. Stratifying the sample by genetic risk revealed some differences in socioeconomic factors and body weight. The high-risk group contained a lower percentage of participants with the highest income and educational attainment. This group also had a mean BMI of 27.5 kg/m^2^, with 24.9% obese, compared to a mean BMI of 26.4 kg/m^2^ and 18.5% obesity for the low BMI-GRS group. Descriptive statistics stratified by quartiles of fast-food outlet exposure are shown in Additional file 1: Table S3, with no systematic differences in mean BMI-GRS observed across fast-food exposure groups.

**Table 1:** Characteristics of participants in the Fenland Study (*n* = 10,798), Cambridgeshire, UK, overall and stratified by genetic risk score for BMI (BMI-GRS)

	BMI-GRS^a^		All (*n* = 10,798)
	Low (*n* = 5399)	High (*n* = 5399)	
Mean BMI-GRS (SD)	2.2 (0.1)	2.4 (0.1)	2.3 (0.2)
Mean age, years (SD)	48.6 (7.4)	48.6 (7.5)	48.6 (7.5)
Men (*n* (%) of participants)	2548 (47.2)	2534 (46.9)	5082 (47.1)
Household income > £40,000 (*n* (%) of participants)	2781 (51.5)	2710 (50.2)	5491 (50.9)
Educational attainment, > 13 years (*n* (%) of participants)	1858 (34.4)	1763 (32.7)	3621 (33.5)
Car access, yes (*n* (%) of participants)	5065 (93.8)	5079 (94.1)	10,144 (93.9)
Health behaviours			
Current or ex-smoker (*n* (%) of participants)	2411 (44.7)	2584 (47.9)	4995 (46.3)
Mean physical activity energy expenditure, kJ/kg/day (SD)	53.8 (22.0)	54.0 (22.1)	53.9 (22.0)
Food environment exposures^b^			
Mean supermarket availability (SD)	2.1 (3.1)	2.0 (2.9)	2.0 (3.0)
Mean fast-food outlet availability (SD)	9.0 (11.7)	8.5 (11.2)	8.8 (11.4)
Crude anthropometric outcomes			
Mean body mass index, kg/m^2^ (SD)	26.4 (4.6)	27.5 (5.0)	26.9 (4.8)
Overweight, 25 ≥ BMI < 30 kg/m^2^ (*n* (%) of participants)	2101 (38.9)	2217 (41.1)	4318 (40.0)
Obese, BMI ≥ 30 kg/m^2^ (*n* (%) of participants)	1001 (18.5)	1344 (24.9)	2345 (21.7)
Adjusted anthropometric outcomes			
Body mass index, *β* (95% CI)			
Model 1^c^	REF	1.11 (0.93, 1.29)**	–
Model 2^d^	REF	1.06 (0.89 1.23)**	–
Overweight, 25 kg/m^2^ ≥ BMI < 30 kg/m^2^, RR (95% CI)			
Model 1^c^	REF	1.34 (1.23, 1.46)**	–
Model 2^d^	REF	1.34 (1.23, 1.47)**	–
Obese, BMI ≥ 30 kg/m^2^, RR (95% CI)			
Model 1^c^	REF	1.69 (1.53, 1.88)**	–
Model 2^d^	REF	1.73 (1.55, 1.93)**	–

**P < 0.001

^a^BMI-GRS, two groups split by sample median: low ≤ 2.29; high > 2.29

^b^Based on counts of food outlets in home neighbourhoods

^c^Model 1 adjusted for age and sex

^d^Model 2 additionally adjusted for household income, highest educational attainment, car access, smoking status, physical activity energy expenditure, counts of supermarkets in home neighbourhoods, and counts of fast-food outlets in home neighbourhoods

### BMI and weight status according to genetic predisposition to obesity

High genetic risk of obesity was positively associated with greater BMI, risk of overweight and obesity (Table 1). In model 1 (adjusted for age and sex), the high BMI-GRS group had 1.11 kg/m^2^ (95% CI, 0.93 to 1.29) higher BMI, had 1.34 (95% CI, 1.23 to 1.46) times greater risk of overweight, and 1.69 (95% CI, 1.53 to 1.88) times greater risk of obesity, than the low BMI-GRS group. These associations were robust to further adjustment for socioeconomic, behavioural and neighbourhood-level covariates (model 2), including neighbourhood fast-food outlet exposure; those at higher genetic risk maintained a 1.06kg/m^2^ (95% CI, 0.89 to 1.23) higher BMI, had 1.34 (95% CI, 1.23 to 1.47) times greater risk of overweight, and 1.73 (95% CI, 1.55 to 1.93) times greater risk of obesity.

### BMI and weight status according to fast-food outlet exposure

Higher neighbourhood fast-food outlet exposure was positively associated with greater BMI and risk of obesity, with a suggestion of a dose-response. After adjustment for BMI-GRS alongside demographic, socioeconomic, and behavioural covariates, those most exposed to fast-food outlets (Q4) had on average 0.65 kg/m^2^ (95% CI, 0.23 to 1.06) higher BMI than those least exposed (Table 2). Those most exposed also had 1.58 (95% CI, 1.21 to 2.05) times greater risk of obesity.

**Table 2:** Associations between fast-food outlet exposure and each of body mass index (also stratified by genetic risk score for BMI (BMI-GRS)) and risks of overweight and obesity in the Fenland Study (*n* = 10,798), Cambridgeshire, UK, estimated using linear and multinomial logistic regression models, respectively

	Per five additional neighbourhood fast-food outlets^a^	Quartiles (Q) of fast-food outlet exposure^b^			
		Q1 (*n* = 4167)	Q2 (*n* = 1360)	Q3 (*n* = 3096)	Q4 (*n* = 2175)
Body mass index, *β* (95% CI)^c^	0.14 (0.05, 0.23)*	REF	− 0.08 (− 0.36, 0.20)	0.27 (0.04, 0.50)*	0.65 (0.23, 1.06)*
BMI-GRS low (*n* = 5399)^d^	0.24 (0.12, 0.36)*	REF	0.08 (− 0.30, 0.46)	0.30 (− 0.01, 0.61)	1.10 (0.54, 1.66)*
BMI-GRS high (*n* = 5399)^d^	0.06 (− 0.07, 0.19)	REF	− 0.26 (− 0.67, 0.16)	0.28 (− 0.06, 0.62)	0.18 (− 0.44, 0.79)
Overweight, 25 kg/m^2^ ≥ BMI < 30 kg/m^2^, RR (95% CI)^c^	1.02 (0.98, 1.07)	REF	0.96 (0.83, 1.11)	1.07 (0.95, 1.21)	1.04 (0.84, 1.30)
Obese, BMI ≥ 30 kg/m^2^, RR (95% CI)^c^	1.08 (1.02, 1.14)*	REF	0.94 (0.79, 1.13)	1.19 (1.21, 2.05)*	1.58 (1.21, 2.05)*

*P < 0.05

Interaction (fast-food outlet exposure quartiles x genetic risk score for BMI (BMI-GRS) *z*-scores) tested using an *F*-test for BMI and a likelihood ratio test for overweight and obesity: BMI *P* = 0.09; overweight *P* = 0.51; obesity *P* = 0.27

For the overweight and obese outcomes, estimates of the association are from multinomial logistic regression models and hence are risk ratios (relative to normal weight)

^a^Associations with BMI, overweight and obesity per five additional neighbourhood fast-food outlets are shown in addition to estimates per quartile, which is our primary operationalisation of fast-food outlet exposure

^b^Home neighbourhood fast-food outlet exposure, quartiles (Q): Q1 (least exposed) = 0–1 outlets; Q2 = 2; Q3 = 3–14; Q4 (most exposed) = 15–51. Quartiles are unequal in sample size due to the distribution of the underlying data

^c^Adjusted for age, sex, household income, highest educational attainment, car access, smoking status, physical activity energy expenditure, counts of supermarkets in home neighbourhoods, and BMI-GRS

^d^Results from a model stratified by sample median BMI-GRS; adjusted as for other models but omitting adjustment for BMI-GRS

### BMI and weight status according to genetic predisposition to obesity and fast-food outlet exposure

**Fig. 1:**
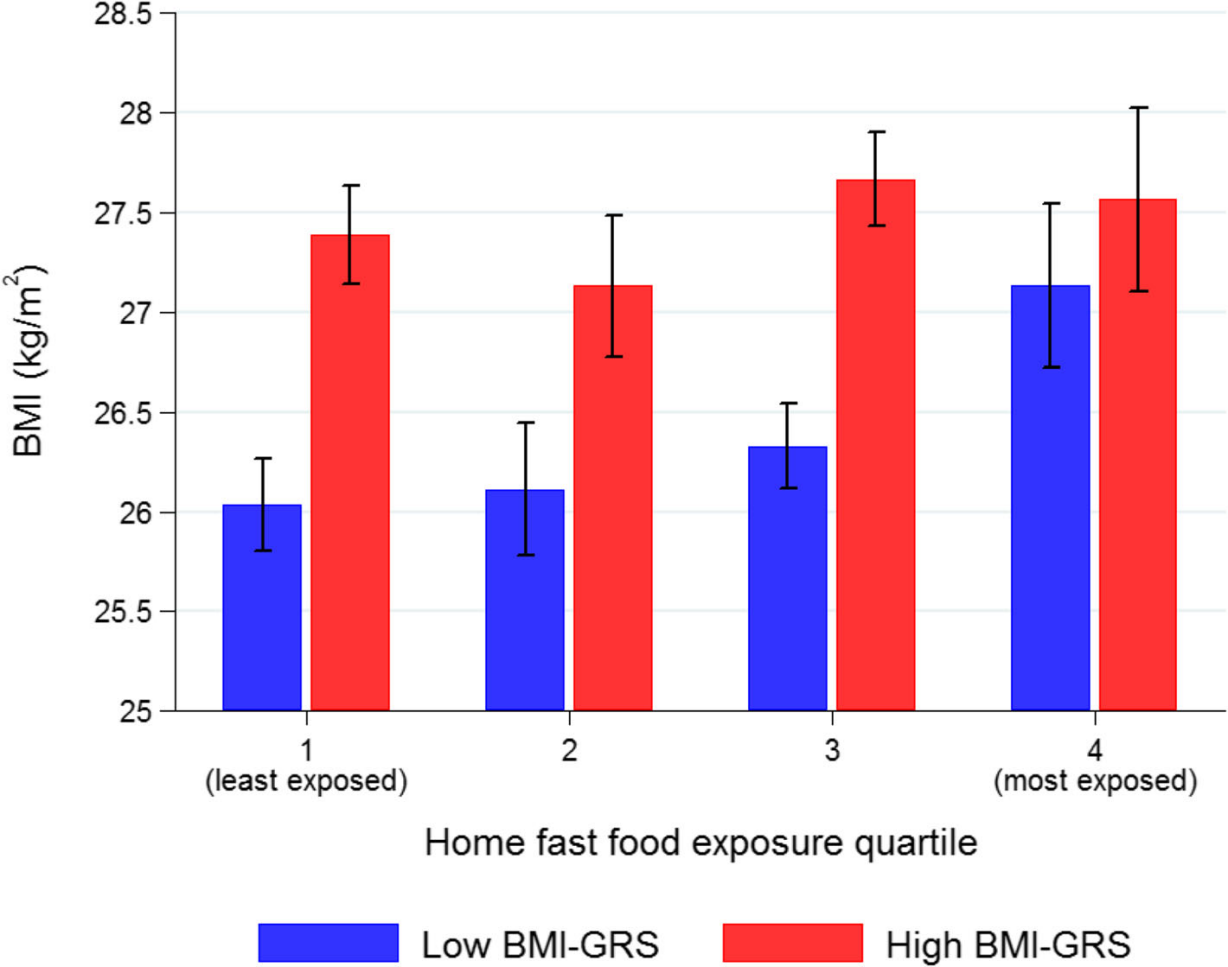
Mean (95% CI) BMI per quartile of fast-food outlet exposure in the Fenland Study (*n* = 10,798), stratified by genetic risk score for BMI (BMI-GRS, split by sample median: low ≤ 2.29; high > 2.29), adjusted for age, sex, household income, highest educational attainment, car access, smoking status, physical activity energy expenditure, counts of supermarkets in home neighbourhoods

There was no evidence of interaction between BMI-GRS and fast-food outlet exposure on BMI (*P* = 0.09), the risk of being overweight (*P* = 0.51), or obese (*P* = 0.27). In an adjusted model stratified by BMI GRS (Table 2), those in the low genetic obesity risk group who were most exposed to fast-food outlets (Q4) had a significantly higher BMI (1.10 kg/m^2^, 95% CI, 0.54 to 1.66). In the high genetic obesity risk group, those most exposed to fast-food outlets did not have a significantly higher BMI (0.18 kg/m^2^, 95% CI, − 0.44 to 0.79). Adjusted means and 95% CIs for BMI in high and low genetic risk groups are shown in Fig. 1.

High BMI-GRS in combination with highest fast-food outlet exposure was associated with significantly greater risk of obesity (RR = 2.70, 95% CI, 1.99 to 3.66), relative to those at low genetic predisposition and least exposed to fast-food outlets (Table 3).

**Table 3:** Combined associations of fast-food outlet exposure and genetic risk score for BMI (BMI-GRS) with risk of obesity in the Fenland Study (*n* = 10,798), Cambridgeshire, UK, estimated using multinomial logistic regression with a single reference group. Overweight results shown in supplementary materials

	Quartiles (Q) of fast-food outlet exposure^a^							
	Q1 (*n* = 4167)		Q2 (*n* = 1360)		Q3 (*n* = 3096)		Q4 (*n* = 2175)	
BMI-GRS^b^	Obese/normal weight (*n*), % obese^c^	RR (95% CI)	Obese/normal weight (*n*), % obese^c^	RR (95% CI)	Obese/normal weight (*n*), % obese^c^	RR (95% CI)	Obese/normal weight (*n*), % obese^c^	RR (95% CI)
Low (*n* = 5399)	392/836, 19.1	REF	122/286, 18.2	1.08^d,^^e^ (0.83, 1.40)	308/620, 19.9	1.21^d,^^e^ (0.99, 1.48)	179/555, 16.0	1.73^d,^^e^ (1.27, 2.35)**
High (*n* = 5399)	559/682, 26.5	1.84^d,^^e^ (1.55, 2.19)**	158/247, 22.9	1.54^d,^^e^ (1.20, 1.98)*	413/493, 26.7	2.20^d,^^e^ (1.81, 2.68)**	214/416, 20.3	2.70^d,^^e^ (1.99, 3.66)**

*P < 0.05; **P < 0.001

^a^Home neighbourhood fast-food outlet exposure, quartiles (Q): Q1 (least exposed) = 0–1 outlets; Q2 = 2; Q3 = 3–14; Q4 (most exposed) = 15–51. Quartiles are unequal in sample size due to the distribution of the underlying data

^b^BMI-GRS, two groups split by sample median: low ≤ 2.29; high > 2.29

^c^Percent obese as a proportion of all participants, including those normal weight, overweight, and obese

^d^Adjusts for age, sex, household income, highest educational attainment, car access, smoking status, physical activity energy expenditure, counts of supermarkets in home neighbourhoods

^e^RRs relative to a single reference group (REF): those least exposed to fast-food outlets (Q1) and at lower BMI-GRS (low)

## Discussion

In a sample of nearly 11,000 UK adults, we found that genetic predisposition to obesity and neighbourhood fast-food outlet exposure were independently associated with higher BMI and risk of obesity. We did not find evidence that the association of fast-food outlet exposure with BMI, overweight, or obesity differed between the low and high genetic risk groups. In combination, we observed a nearly threefold risk of obesity in those with both high genetic risk and with highest fast-food outlet exposure, relative to those at low genetic risk and least exposed to fast-food outlets.

The mechanisms by which BMI-associated SNPs confer a greater risk of obesity are not fully elucidated, but two SNPs included in our genetic risk score for BMI, *FTO* and *MC4R*, have been linked to dysregulation of appetite and loss of control over eating [29–32], and preferences towards energy-dense [33], high-fat foods [23, 34]. As such, studies using genetic risk scores similar to the one used here have found that the association between these scores and obesity can be partly explained by appetite and eating behaviours [26, 35]. However, previous research has been limited to examining the interaction of BMI-associated SNPs with behavioural factors (gene-lifestyle interactions) such as diet and physical activity. For example, the influence of *FTO* on obesity risk can be moderated by physical activity [27]. Similarly, dietary risk factors for obesity, including consumption of sugar-sweetened beverages [36], and fried food [37], have been more strongly associated with weight gain in those with higher genetic obesity predisposition.

**Fig. 2:**
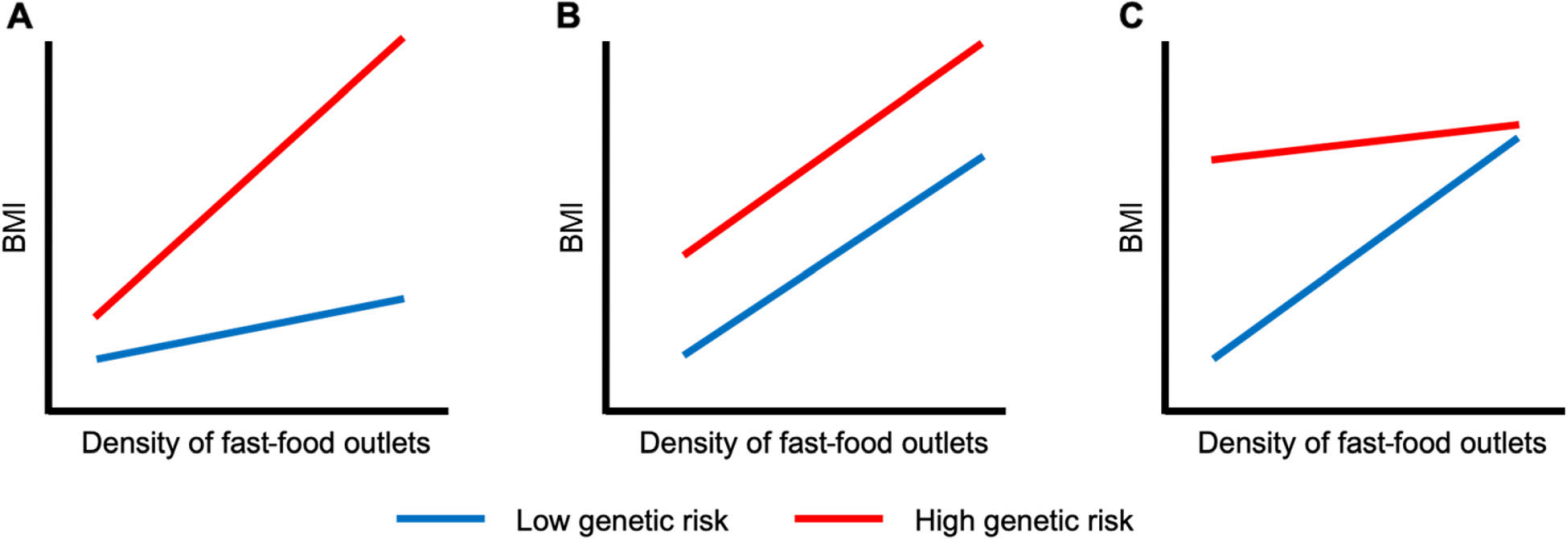
Hypothetical associations between density of (or exposure to) fast-food outlets and BMI, which are: **a** moderated by genetic obesity risk, with the higher genetic risk group showing a stronger environmental association than the lower genetic risk group; **b** not moderated by genetic obesity risk; and **c** moderated by genetic obesity risk, with the higher genetic risk group showing a weaker environmental association than the lower genetic risk group

To our knowledge, this is the first published investigation of the interplay between any characteristic of the *neighbourhood* environment and genetic obesity risk. Our study was motivated to explore the hypothesis that genetic obesity risk would be exaggerated in permissive neighbourhood environments, characterised by an abundance of fast-food outlets, where large portions of affordable, energy-dense foods are readily accessible [38]. We expected that the strength of the relationship between fast-food outlet density and body weight would be *stronger* for those more genetically susceptible to obesity (Fig. 2a). We found no evidence to support this hypothesis. Alternatively, if it were the case that those with higher genetic risk were *equally* susceptible to the food environment, we would have expected the relationship between fast-food outlet density and body weight to be similar in both groups, albeit with those at higher risk having higher body weight at any given level of environmental exposure (Fig. 2b). However, although we found no evidence of interaction, our results indicated a *possible* third alternative, of a *weaker* association of the food environment with BMI among those with higher genetic risk of obesity, compared with those with lower genetic risk (Fig. 2c). We observed that, at highest levels of fast-food outlet exposure, mean BMI for the lower and higher genetic risk groups converged.

The key implication of our findings is the necessity for further research to test our observations and understand mechanisms in other samples. It is possible, for example, that the contribution of the food environment may simply be less detectable among a population of adults with higher risk of obesity stemming from multiple biological and metabolic determinants [25]. This explanation would be consistent with Rothman’s component cause model, which suggests that for outcomes with multiple causes, the apparent strength of any single causal component is influenced by the relative prevalence of other component causes [39]. Nonetheless, in the immediate term and over and above the contribution of genetic susceptibility, neighbourhood fast-food outlet exposure appears to be a significant and importantly modifiable contributor, subject to change via urban planning (‘zoning’) as a structural intervention to prevent obesity and promote the public’s health [40].

The major strengths of this study include objective assessment of neighbourhood fast-food outlet exposure, using accurate and contemporaneous food outlet location data; objective height and weight data, measured by trained researchers; and use of an empirically-derived genetic risk score based on 96 BMI-associated SNPs. The main limitation of this study is the cross-sectional, observational study design, which limits causal inference. It is possible that preference for fast food may drive the selection of residential neighbourhoods with abundant fast-food outlet access (reverse causality). Our estimates of combined associations might be confounded because preference for energy-dense fast food may arise as a function of genetic obesity predisposition. Our fast-food outlet exposure models may have residual confounding, for example from unobserved environmental attributes, such as exposure to food outlets beyond the home neighbourhood. This said, our models were comprehensively adjusted for behavioural and sociodemographic covariates.

Some misclassification may have occurred as our exposure (number of fast-food outlets) and outcome (adiposity) were measured at different time points (2011 and 2005–2015, respectively). This risk was minimised as far as possible through exposure data being collected at approximately the mid-point of the period of outcome data collection. Moreover, we minimised misclassification through operationalising fast-food outlet exposure in quartiles, which would have been less sensitive to food environment change over time. To test this, we cross-classified all participants, comparing their exposure classification based on 2011 food environment data, against that based on food environment data from 2014 (courtesy of the Food environment assessment tool, powered by Ordnance Survey) [41]. The resulting cross-classification was high (*r*_*s*_ = 0.94, *P* < 0.001). Therefore, any individual recruited into the Fenland Study between 2011 and 2014 would likely have been characterised similarly in terms of their fast-food outlet exposure. We have described the limitations of our neighbourhood fast-food outlet exposure metric previously [19, 22, 24].

We used a genetic risk score composed of 96 BMI-increasing alleles, which enabled us to detect a large main effect both independently and in combination with neighbourhood fast-food outlet exposure. However, this approach masks the contribution of individual SNPs, which may act through discrete behavioural or metabolic pathways of relevance to the food environment. Moreover, given the highly polygenic nature of body weight, genetic risk scores that use a genome-wide set of common variants in their construction may have stronger predictive power for obesity and BMI and should be considered for future research [42]. The Fenland Study is a population-based cohort study, with working-age adults recruited who were largely educated, employed and white British, living in Cambridgeshire, which is a county in the East of England comprising urban, suburban and rural areas, including the major cities of Cambridge and Peterborough. Characteristics of this population and study area, although common elsewhere in the UK and beyond, may influence the generalisability of our findings.

## Conclusions

Our study confirms previously-identified associations of both genetic and neighbourhood risk factors with adiposity, and for the first time demonstrated independence in these relationships. These important drivers of the obesity epidemic have to date been studied in isolation. Although obesity-related SNPs contribute substantially to obesity in the population, neighbourhood fast-food outlet exposure also appears to be a significant determinant and an important and modifiable target of policy intervention to prevent obesity and promote the public’s health. We found no evidence of interaction between these determinants.

## Supplementary Information

**Additional file 1: Table S1.** Detailed fast-food outlet and chain supermarket characteristics. **Table S2.** Fenland Study cohort, analytic sample and excluded subset sociodemographic comparisons. **Table S3.** Characteristics of participants in the Fenland Study (*n* = 10,798), Cambridgeshire, UK, overall and stratified by quartile of exposure to fast-food outlets. **Table S4.** Associations between genetic risk score (BMI-GRS) and each of: body mass index and risks of overweight and obesity in the Fenland Study (n = 10,798), Cambridgeshire, UK, estimated using linear and multinomial logistic regression models, respectively. **Table S5.** Combined associations of fast-food outlet exposure and genetic risk score (BMI-GRS) with risk of overweight in the Fenland Study (n = 10,798), Cambridgeshire, UK, estimated using multinomial logistic regression with a single reference group. **Fig. S1.** Flow diagram for Fenland Study cohort sample restriction to the Fenland Study analytic sample.

## Data Availability

The Fenland Study data analysed here are not publicly available for reasons of confidentiality, but may be available upon reasonable request. For data sharing enquiries, please email datasharing@mrc-epid.cam.ac.uk.

## Abbreviations

BMI: Body mass index
CI: Confidence interval
GRS: Genetic risk score
GWAS: Genome-wide association study
Q: Quartile
RR: Relative risk
SNP: Single nucleotide polymorphism
UK: United Kingdom
