# Supplementary material for: Independent and combined associations between fast-food outlet exposure and genetic risk for obesity: a population-based, cross-sectional study in the UK
Source: BMC Med. 2021 Feb 15;19:49. doi: 10.1186/s12916-021-01902-z (PMC7885578; doi:10.1186/s12916-021-01902-z)
Supplement: Supplementary file 1 — Additional file 1: Table S1. Detailed fast-food outlet and chain supermarket characteristics. Table S2. Fenland Study cohort, analytic sample and excluded subset sociodemographic comparisons. Table S3. Characteristics of participants in the Fenland Study (n = 10,798), Cambridgeshire, UK, overall and stratified by quartile of exposure to fast-food outlets. Table S4. Associations between genetic risk score (BMI-GRS) and each of: body mass index and risks of overweight and obesity in the Fenland Study (n = 10,798), Cambridgeshire, UK, estimated using linear and multinomial logistic regression models, respectively. Table S5. Combined associations of fast-food outlet exposure and genetic risk score (BMI-GRS) with risk of overweight in the Fenland Study (n = 10,798), Cambridgeshire, UK, estimated using multinomial logistic regression with a single reference group. Fig. S1. Flow diagram for Fenland Study cohort sample restriction to the Fenland Study analytic sample. [file 12916_2021_1902_MOESM1_ESM.docx]

**Additional file 1**

Independent and combined associations between fast-food outlet exposure and genetic risk for obesity: a population-based, cross-sectional study in the UK

Thomas Burgoine, Pablo Monsivais, Stephen J Sharp, Nita G Forouhi, Nicholas J Wareham

**Table S1:** Detailed fast-food outlet and chain supermarket characteristics

| **Business type** | **Characteristics** |
| --- | --- |
| Fast-food outlet | Hot food sold.  Food ordered and paid for at the till.  No wait staff.  No or limited, informal seating options for dining in. |
| Chain supermarket | Departmentalised, self-service store selling a wide range of groceries and household goods.  Belonging to a major UK supermarket chain, who between them share 88% of the UK grocery market: Tesco, Sainsbury’s, ASDA, Morrisons, Waitrose, Aldi and Co-operative stores. |

**Fig. S1:** Flow diagram for Fenland Study cohort sample restriction to the Fenland Study analytic sample

**Fenland Study cohort** (*n* = 12,435)

Missing, incomplete or not geocodeable home address, or home address outside study area: *n* = 35

Missing genetic data: *n* = 956

Missing or incomplete household income, highest education, car access, smoking status, physical activity energy expenditure, or body mass index data: *n* = 646

**Fenland Study analytic sample** (*n* = 10,798)

**Table S2:** Fenland Study cohort, analytic sample and excluded subset sociodemographic comparisons

|  | Fenland Study cohort (*n* = 12,435) ^a^ | Fenland Study analytic sample (*n* = 10,798) | Fenland Study excluded subset (*n* = 1637) ^a^ |
| --- | --- | --- | --- |
| Mean age, years (SD) | 48.6 (7.5) | 48.6 (7.5) | 48.5 (7.8) |
| Sex, men (%) | 46.2 | 47.1 | 40.4 |
| Mean BMI (SD), kg/m^2^ | 26.9 (4.8) | 26.9 (4.8) | 26.8 (5.9) |
| Weight status, obese (%) ^b^ | 21.8 | 21.7 | 22.0 |
| Smoking status, current or ex (%) | 45.9 | 46.3 | 46.2 |
| Highest education, Higher (%) ^c^ | 33.9 | 33.6 | 36.1 |
| Car access, yes (%) | 93.3 | 93.9 | 94.0 |
| ^a^ Data summarised for variables with < 1% missingness  ^b^ Body mass index ≥ 30kg/m^2^  ^c^ Participants completing > 13 years of education | | | |

| **Table S3:** Characteristics of participants in the Fenland Study (*n* = 10,798), Cambridgeshire, UK, overall and stratified by quartile of exposure to fast-food outlets | | | | | |
| --- | --- | --- | --- | --- | --- |
|  | Quartiles (Q) of fast-food outlet exposure ^a^ | | | | All  (*n* = 10,798) |
|  | Q1 (*n* = 4167) | Q2 (*n* = 1360) | Q3 (*n* = 3096) | Q4 (*n* = 2175) |  |
| Mean BMI-GRS (SD) | 2.3 (0.2) | 2.3 (0.2) | 2.3 (0.2) | 2.3 (0.2) | 2.3 (0.2) |
| Mean age, years (SD) | 49.0 (7.2) | 48.7 (7.5) | 47.7 (7.7) | 49.0 (7.6) | 48.6 (7.5) |
| Men (*n* (%) of participants) | 1916 (46.0) | 627 (46.1) | 1465 (47.3) | 1074 (49.4) | 5082 (47.1) |
| Household income > £40,000 (*n* (%) of participants) | 2153 (51.7) | 798 (58.7) | 1435 (46.4) | 1105 (50.8) | 5491 (50.9) |
| Educational attainment, > 13 years (*n* (%) of participants) | 1170 (28.1) | 462 (34.0) | 923 (29.8) | 1066 (49.0) | 3621 (33.5) |
| Car access, yes (*n* (%) of participants) | 4088 (98.1) | 1322 (97.2) | 2870 (92.7) | 1864 (85.7) | 10,144 (93.9) |
| *Health behaviours* |  |  |  |  |  |
| Current or ex-smoker (*n* (%) of participants) | 1853 (44.5) | 635 (46.7) | 1462 (47.2) | 1045 (48.1) | 4995 (46.3) |
| Mean physical activity energy expenditure, kJ/kg/day (SD) | 54.9 (22.8) | 53.2 (21.7) | 52.6 (21.8) | 54.2 (21.1) | 53.9 (22.0) |
| *Food environment exposures* ^b^ |  |  |  |  |  |
| Mean supermarket availability (SD) | 0.0 (0.2) | 1.1 (0.8) | 1.8 (1.2) | 6.8 (3.4) | 2.0 (3.0) |
| Mean fast-food outlet availability (SD) | 0.4 (0.5) | 2.0 (0.0) | 9.2 (4.1) | 28.4 (8.8) | 8.8 (11.4) |
| *Crude anthropometric outcomes* |  |  |  |  |  |
| Mean body mass index, kg/m^2^ (SD) | 27.1 (4.8) | 26.8 (4.6) | 27.2 (5.0) | 26.3 (4.7) | 26.9 (4.8) |
| Overweight, 25 ≥ BMI < 30kg/m^2^ (*n* (%) of participants) | 1698 (40.8) | 547 (40.2) | 1262 (40.8) | 811 (37.3) | 4318 (40.0) |
| Obese, BMI ≥ 30kg/m^2^ (*n* (%) of participants) | 951 (22.8) | 280 (20.6) | 721 (23.3) | 393 (18.1) | 2345 (21.7) |
| ^a^ Home neighbourhood fast-food outlet exposure, quartiles (Q)  ^b^ Based on counts of food outlets in home neighbourhoods | | | | | |

**Table S4:** Associations between genetic risk score (BMI-GRS) and each of: body mass index and risks of overweight and obesity in the Fenland Study (*n* = 10,798), Cambridgeshire, UK, estimated using linear and multinomial logistic regression models, respectively.

|  | Per one standard deviation greater BMI-GRS ^a^ |
| --- | --- |
|  |  |
| Body mass index, *β* (95% CI) ^b^ | 0.65 (0.56, 0.73)** |
| Overweight, 25 kg/m^2^ ≥ BMI < 30kg/m^2^, RR (95% CI) ^b^ | 1.20 (1.15, 1.26)** |
| Obese, BMI ≥ 30kg/m^2^, RR (95% CI) ^b^ | 1.41 (1.33, 1.49)** |
| ** *P* < 0.001.  ^a^ Associations with BMI, overweight and obesity per one standard deviation greater BMI-GRS  ^b^ Adjusted for household income, highest educational attainment, car access, smoking status, physical activity energy expenditure, counts of supermarkets in home neighbourhoods and counts of fast-food outlets in home neighbourhoods. | |

| **Table S5:** Combined associations of fast-food outlet exposure and genetic risk score (BMI-GRS) with risk of overweight in the Fenland Study (*n* = 10,798), Cambridgeshire, UK, estimated using multinomial logistic regression with a single reference group | | | | | | | | | |  |
| --- | --- | --- | --- | --- | --- | --- | --- | --- | --- | --- |
|  | Quartiles (Q) of fast-food outlet exposure ^a^ | | | | | | | | |  |
|  | Q1 (*n* = 4167) | | Q2 (*n* = 1360) | | Q3 (*n* = 3096) | | | Q4 (*n* = 2175) | | |
| BMI-GRS ^b^ | overweight/normal weight (*n*), % overweight ^c^ | RR (95% CI) | overweight/normal weight (*n*), % overweight ^c^ | RR (95% CI) | | overweight/normal weight (*n*), % overweight ^c^ | RR (95% CI) | overweight/normal weight (*n*), % overweight ^c^ | RR (95% CI) |  |
| Low (*n* = 5399) | 829/836, 40.3 | REF | 262/286, 39.1 | 0.99 ^d.e^ | | 623/620, 40.2 | 1.06 ^d,e^ | 387/555, 34.5 | 1.01 ^d,e^ |  |
|  |  |  |  | (0.81, 1.22) | |  | (0.90, 1.24) |  | (0.78, 1.29) |  |
| High (*n* = 5399) | 869/682, 41.2 | 1.34 ^d,e^ | 285/247, 41.3 | 1.22 ^d,e^ | | 639/493, 41.4 | 1.45 ^d,e^ | 424/416, 40.2 | 1.42 ^d,e^ |  |
|  |  | (1.16, 1.54)** |  | (1.00, 1.50) | |  | (1.23, 1.71)** |  | (1.11, 1.82)* |  |
| * *P* < 0.05; ** P < 0.001  ^a^ Home neighbourhood fast-food outlet exposure, quartiles (Q): Q1 (least exposed) = 0-1 outlets; Q2 = 2; Q3 = 3-14; Q4 (most exposed) = 15-51. Quartiles are unequal in sample size due to the distribution of the underlying data  ^b^ BMI-GRS, two groups split by sample median: Low ≤ 2.29; High > 2.29  ^c^ Percent overweight as a proportion of all participants, including those normal weight, overweight and obese  ^d^ Adjusts for age, sex, household income, highest educational attainment, car access, smoking status, physical activity energy expenditure, counts of supermarkets in home neighbourhoods  RRs relative to a single reference group (REF): those least exposed to fast food outlets (Q1) and at lowest BMI-GRS (low) | | | | | | | | | |  |
